# Individual Participant Data Meta-Analysis for a Binary Outcome: One-Stage or Two-Stage?

**DOI:** 10.1371/journal.pone.0060650

**Published:** 2013-04-09

**Authors:** Thomas P. A. Debray, Karel G. M. Moons, Ghada Mohammed Abdallah Abo-Zaid, Hendrik Koffijberg, Richard David Riley

**Affiliations:** 1 Julius Center for Health Sciences and Primary Care, University Medical Center Utrecht, Utrecht, The Netherlands; 2 European Centre for Environment and Human Health, Peninsula College of Medicine and Dentistry, University of Exeter, at the Knowledge Spa, Royal Cornwall Hospital, Truro, Cornwall, United Kingdom; 3 School of Health and Population Sciences, and School of Mathematics, Public Health, Epidemiology and Biostatistics, College of Medical and Dental Sciences, University of Birmingham, Birmingham, United Kingdom; Universidad Peruana de Ciencias Aplicadas (UPC), Peru

## Abstract

**Background:**

A fundamental aspect of epidemiological studies concerns the estimation of factor-outcome associations to identify risk factors, prognostic factors and potential causal factors. Because reliable estimates for these associations are important, there is a growing interest in methods for combining the results from multiple studies in individual participant data meta-analyses (IPD-MA). When there is substantial heterogeneity across studies, various random-effects meta-analysis models are possible that employ a one-stage or two-stage method. These are generally thought to produce similar results, but empirical comparisons are few.

**Objective:**

We describe and compare several one- and two-stage random-effects IPD-MA methods for estimating factor-outcome associations from multiple risk-factor or predictor finding studies with a binary outcome. One-stage methods use the IPD of each study and meta-analyse using the exact binomial distribution, whereas two-stage methods reduce evidence to the aggregated level (e.g. odds ratios) and then meta-analyse assuming approximate normality. We compare the methods in an empirical dataset for unadjusted and adjusted risk-factor estimates.

**Results:**

Though often similar, on occasion the one-stage and two-stage methods provide different parameter estimates and different conclusions. For example, the effect of erythema and its statistical significance was different for a one-stage (OR = 1.35, 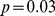) and univariate two-stage (OR = 1.55, 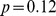). Estimation issues can also arise: two-stage models suffer unstable estimates when zero cell counts occur and one-stage models do not always converge.

**Conclusion:**

When planning an IPD-MA, the choice and implementation (e.g. univariate or multivariate) of a one-stage or two-stage method should be prespecified in the protocol as occasionally they lead to different conclusions about which factors are associated with outcome. Though both approaches can suffer from estimation challenges, we recommend employing the one-stage method, as it uses a more exact statistical approach and accounts for parameter correlation.

## Introduction

A fundamental aspect of epidemiological studies concerns the estimation of associations between independent variables (factors) and dependent variables (outcomes). Outcomes may include such as disease onset, disease presence (diagnosis), disease progression (prognosis), and death. Independent variables may include potential causal factors to unravel the pathophysiology or causal pathway of the outcome under study, but also non-causal predictors or risk-indicators of the outcome to enhance timely detection or prediction of the outcome, perhaps as part of a risk prediction model [1]–[3]. Studies that aim to explore which causal factors or predictors – often out of a number of candidate factors – are independently associated with a particular outcome have been referred to as risk factor or predictor finding studies [3]–[8]. Reliable estimates of such factor-outcome associations are essential, certainly when they are meant to be causal, to properly guide public health initiatives and clinical practice for informing diagnosis and prognosis. As such, primary studies to identify causal factors or predictors are abundant in the medical literature. For example, in patients with neuroblastoma, a review identified 260 primary studies evaluating one or more novel tumour markers for their association with outcome [8]–[10]. When reviewing such evidence across multiple studies, the estimated factor-outcome associations across studies may be inconsistent and even contradictory [11]–[13]. This emphasizes the need for appropriate methods for meta-analysis and evidence synthesis in this area, in order to summarise the factor-outcome associations in the current evidence-base [2], [14]–[20], as commonly applied in intervention research [21]–[25]. However, due to numerous problems of published primary studies investigating factor-outcome associations, especially publication bias and selective reporting, meta-analyses based on published results are notoriously prone to bias [8], [10]. Problems with such aggregate data also arise in clinical research when differential treatment effects by patient characteristics are of concern [26]. Problems with such aggregate data also arise in clinical research when differential treatment effects by patient characteristics are of concern [26].Problems with such aggregate data also arise in clinical research when differential treatment effects by patient characteristics are of concern [26]. Thus there is increasing interest in obtaining individual participant data (IPD) from these studies to facilitate a more reliable meta-analysis.

When IPD are available, meta-analysis is usually performed using a two-stage approach [24]. Each study is summarized by its factor-outcome association estimate and variance in the first stage, and these aggregate data (AD) are then appropriately combined across studies in the second stage. In this manner, a summary effect size, such as the odds or hazard ratio, is produced for each factor-outcome association of interest [27] whilst potentially accounting for between-study heterogeneity (e.g. due to different participant characteristics, methods of measurements, and undergone treatments) [12], [19], [28]–[35]. An alternative method for IPD meta-analysis (IPD-MA) is a one-stage approach which synthesises the IPD from all studies in a single step, whilst accounting for clustering of patients within studies [36]–[38]. Assuming the sufficient AD are obtained from each study for the two-stage method, it is widely believed that one-stage and two-stage methods lead to similar conclusions [39]–[41]; however, empirical comparisons are relatively few. Indeed, because the design and implementation of one-stage and two-stage random-effects models may substantially differ, it is important to ascertain whether the choice of method can influence the final conclusions about whether a factor has a (statistically) significant association with the outcome.

In a recent empirical evaluation using a meta-analysis of 24 randomised trials of antiplatelets to prevent preeclampsia, Stewart *et al.* [37] conclude that ‘two-stage and one-stage approaches to analysis produce similar results’ and ‘where an IPD review evaluates effectiveness based on sufficient data from randomised controlled trials, one-stage statistical analyses may not add much value to simpler two-stage approaches’. It is important to consider if this recommendation is valid in other empirical examples, and if it translates to epidemiological studies. In particular, epidemiological studies of factor-outcome associations may be affected by several covariates, namely confounders (in causal factor studies) or other predictors (in predictor finding studies) [33], [42], [43]. This situation may also arise in clinical trials when interactions occur between treatment effects and covariates, or when adjustment is needed for prognostic factors that are unbalanced between groups. Thus the random-effects framework needs to accommodate these covariates during modeling in order to estimate factor-outcome associations after adjusting for other factors. Factors that are strongly associated with the outcome might retain their association even when adjusting for other variables. However, there has again been little comparison of one-stage and two-stage IPD-MA methods when adjustment is required [44], [45].

The aim of this article is to describe and empirically evaluate possible one-stage and two-stage IPD-MA models for synthesizing (causal or predictive) factor-outcome association estimates across multiple studies where a continuous or binary factor is of interest in relation to a binary outcome. It is therefore similar in spirit to a recent description of methods for meta-analysis of time-to-event outcomes [46]. The methods are compared using an empirical example, to illustrate their advantages, differences and accessibility. Our methods all assume that between-study heterogeneity in baseline risk and factor-outcome associations exists, as it likely in practice, and so we only consider random-effects IPD-MA models. We examine different assumptions concerning the random effects, and consider how the models can be extended to adjust for other factors. Hereto, we describe two two-stage and three one-stage models for estimating unadjusted and adjusted factors. We finish by depicting some estimation procedures and approximations, and conclude with discussion and recommendations.

## Motivating Example

Deep Vein Thrombosis (DVT) is a blood clot that forms in a vein in the body (usually in the lower leg or thigh). A (part of such) clot can break off and be carried through the bloodstream to the lungs and there cause a blockage (pulmonary embolism), preventing oxygenation of the blood and potentially causing death. The diagnosis DVT presence or absence can (ultimately) be made using repeated leg ultrasound, which requires patient referral and is to some extent burdening and time and money consuming. Hence, it is desirable to predict the presence or absence of DVT without having to refer patients for more cumbersome testing, by rather using easy to obtain predictors from their patient history, physical examination and simple blood assays. For this reason, in patients with a suspected DVT various studies aimed at estimating which factors – out of a range of candidate factors – are indeed associated with the presence or absence of DVT; in other words, which factors are useful diagnostic predictors of the probability that a patient truly has DVT.

A previous systematic review collected the IPD of patients with a suspected DVT from 13 studies (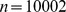), and this IPD contains information about the patients' history, physical examination and results from a biomarker test (Table 1 and Table S1) [47]. In this article, we use these data to illustrate the described meta-analysis methods for identifying important risk factors. We assume random effects for factor-outcome associations as the presence of heterogeneity between studies is expected due to differences in locale, setting and time. Detailed information about the included studies and predictors is available in Table S1 and Table S2.

**Table 1:** Overview of the DVT data.

Study	N	ddimdich = 1	notraum = 1	coag = 1	eryt = 1	sex = 1	malign = 1	par = 1
1	1028(131)	472 (117)	743 (104)	19 (3)	382 (52)	376 (66)	54 (15)	13 (2)
2	814(318)	598 (313)				307(146)	86 (43)	35(17)
3	153 (26)		103 (16)		51(15)	73 (10)	7 (4)	12 (1)
4	1756(411)	910 (387)	1497 (361)	68 (20)		654(192)	224 (84)	101(35)
5	791 (126)	572 (91)	650 (111)	191(31)		301(59)	38 (8)	112(18)
6	1075(190)	424 (161)	857 (158)	52 (17)		471 (97)	55 (25)	50 (11)
7	429 (61)					153 (28)	47 (17)	12 (2)
8	325 (52)	214 (51)		57 (11)		128 (24)	12 (5)	14 (2)
9	1295(289)	897 (276)	1098 (257)			467(137)	81 (34)	178(37)
10	436 (42)			82 (5)		145 (20)	26 (8)	13 (2)
11	541 (121)	266 (108)	373 (92)	14 (4)	144(38)	238 (62)	99 (47)	34 (13)
12	550 (55)					210 (27)	50 (17)	12 (1)
13	809 (42)					324 (21)	55 (10)	27 (5)

		tend = 1	leg = 1	calfdif3 = 1	pit = 1	vein = 1	altdiagn = 1	surg = 1
1	1028(131)	562 (69)	232 (46)	311 (72)	704(108)	155 (28)	669 (22)	81 (11)
2	814 (318)	541 (237)	169 (89)	353 (186)	419(196)	127 (57)	217 (58)	75 (34)
3	153 (26)	82 (19)	51 (20)	59 (19)	73 (22)	25 (8)	74 (3)	24 (10)
4	1756(411)	664 (238)	607 (251)	426 (210)	950(272)	283 (92)	906 (92)	198(77)
5	791 (126)	572 (90)	353 (79)	322 (79)	490 (85)	155 (32)	300 (43)	105(25)
6	1075(190)	494 (118)	217 (67)	303 (106)	357(100)	43 (16)	448 (26)	168(45)
7	429 (61)	203 (41)	30 (13)	96 (33)	87 (24)	33 (5)	176 (17)	25 (6)
8	325 (52)	161 (31)	47 (21)	93 (33)	97 (29)	39 (9)	114 (8)	16 (6)
9	1295(289)	924 (208)	583 (164)	556 (194)	799(193)	257 (82)	782 (98)	181(54)
10	436 (42)	222 (28)	168 (28)	66 (13)	91 (18)	1 (0)	119 (5)	58 (9)
11	541 (121)	239 (74)	152 (67)	162 (63)	270 (86)	38 (16)	313 (22)	96 (34)
12	550 (55)	176 (21)	83 (16)	114 (30)	251 (40)	28 (8)	245 (16)	39 (7)
13	809 (42)	258 (22)	75 (8)	153 (18)	196 (17)	32 (3)	399 (9)	45 (4)

Observed factor level counts (for which dvt = 1) for binary risk factors in each study of the DVT case study. Entries are left blank for studies that did not measure the corresponding factor.

## Methods

This section describes the framework for random-effects IPD-MA modeling of risk factor (predictor finding) studies with a binary outcome. Hereto, it identifies two sources of data: IPD and AD. IPD is represented by patient-level factor values (covariates) and outcomes, whereas AD consists of study-level summaries such as the estimated log odds ratios and corresponding standard errors for the factor-outcome associations reported [48]. We describe two-stage and then one-stage IPD-MA approaches [49] and describe how to account for differences in baseline risk across studies (clustering). Further, we show how to extend these methods to adjust for known risk factors, and evaluate some important estimation difficulties that arise when relatively few data are available. The DVT data is used to illustrate the methods and to identify some important differences.

### Two-stage IPD Methods

#### First stage

In a two-stage method, the IPD are first analyzed separately in each study using an appropriate statistical method for binary outcome data. For example, consider where a single risk factor is of interest, then the logistic regression model is:
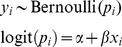
(Model♮_1_)with unknown parameters 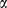 (intercept) and 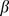 (slope representing the association between factor 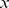 and binary outcome 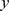). The logit outcome probability for subject 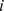, 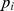, is then a linear function of the factor 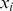. The resulting estimates from study 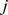 are denoted as 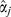 (intercept) and 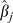 (log odds ratio). Consequently, the first step yields the intercept and the factor-outcome association estimates, and their associated within-study covariance matrix (containing the variance of the intercept 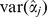 and each association 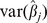, as well as their respective covariances 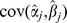) for each individual study. By utilising all the model parameter estimates, their variances and their correlation (covariance), the original IPD is reduced to AD for each study [50], [51]. If IPD are not available, such AD may alternatively be sought from study publications or study authors.In the second stage, this AD from each study are synthesized using a suitable model for meta-analysis of AD [30], [43], [52], with potential options as follows.

**Second Stage. Option 1. Full (bivariate) meta-analysis AD model:** The AD are combined by a bivariate random-effects model that simultaneously synthesises the factor-outcome association (beta) estimates and the baseline risk (intercept) estimates whilst accounting for their correlation. The model assumes that the true underlying effect of the 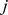
th study (asymptotically) arises from a multivariate normal (MVN) distribution [53], and incorporates within- and between-study covariance. Specifically, the model fits the following marginal distributions:
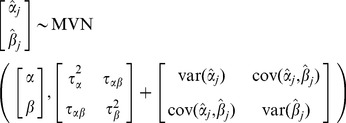
(Model1)with unknown parameters 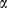, 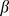, 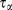, 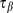 and 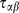. Here, 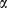 and 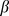 represent the *average* baseline risk and factor-outcome association across studies, respectively, 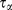 and 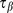 describe their respective degree of heterogeneity between studies, and 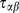 their between-study covariance.

**Option 2. Traditional (univariate) meta-analysis AD model:** Most researchers ignore within-study and between-study covariances in parameter estimates and thus assume that 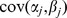 and 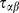 equal 0 [51]. Essentially, this reduces Model 1 to a univariate meta-analysis of the factor-outcome association, and is similar to the commonly applied DerSimonian and Laird's classical random-effects meta-analysis model [21], [54], where:
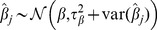
(Model2)with unknown parameters 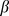 and 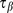. This model no longer synthesises the baseline risk across studies, and just pools the factor-outcome associations.

### One-stage Methods

In a one-stage method, the IPD from all studies are modeled simultaneously whilst accounting for the clustering of subjects within studies. The one-stage IPD-MA framework is a (multilevel) logistic regression model with random effects. Different specifications are possible, as now described.

**Option 1. Fully (bivariate) random-effects one-stage model:** Here, as in Model 1, random effects are specified for both the intercept and the slope, and their between-study covariance is modelled
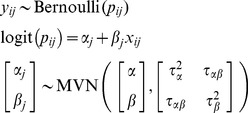
(Model3)where 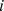 indicates observations at the individual level and 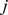 again represents the study level. Note that 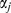 and 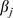 are not explicitly estimated (in contrast to Model 1, where it represents the AD from the individual studies) but follow from the unknown parameters 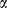, 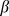, 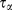, 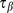 and 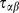. These parameters have the same interpretation as those from Model 1.

**Option 2. Reduced random-effects one-stage model:** In a *reduced* one-stage model, independent random effects are assumed for the intercept and slope in order to avoid estimating the between-study covariance, which can often be problematic:
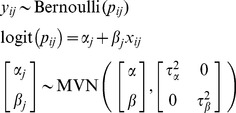
(Model4)


**Option 3. Stratified one-stage model:** Finally, it is possible to reduce the number of assumptions by estimating a *stratified* one-stage model. This model no longer estimates an underlying average for the intercepts but rather estimates a separate intercept for each study. Thus the between-study normality assumption for the intercept term is no longer required for 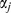, and there is no need to estimate a between-study covariance term. However, heterogeneity in the the factor-outcome association is still modelled using a random effect:
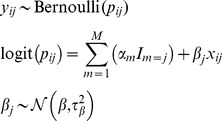
(Model5)where the indicator term 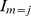 indicates that a separate intercept should be estimated for each study 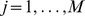. Similar to Model 3 and Model 4, 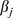 is not explicitly estimated but follows from the unknown parameters 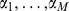, 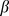 and 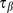.

### Extending the One-stage and Two-stage Models to Examine Multiple Risk Factors

Previously, we described models for summarizing unadjusted factor-outcome associations. Although these models are fairly straightforward to implement, it is well known that factor-outcome associations are often influenced by extraneous variables rendering exposure groups incomparable. This situation may, for instance, arise when associations are estimated from cohort and cross-sectional studies (prognostic research) or treatment-by-patient-characteristic interactions occur (intervention research). In addition, several authors have recommended that each factor should be studied for their incremental (causal or predictive) value beyond established risk factors [55], [56]. This raises the need for multivariable analyses, where the factor-outcome association under investigation is adjusted for potential confounders or other known predictors. Consequently, the methods from previous section performing a univariate (or bivariate) meta-analysis need to be extended to perform a (multivariate) meta-analysis where the factor-outcome associations (and intercept) are adjusted for 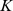 additional factors.

#### Extended two-stage models

For the two-stage method, multivariable logistic regression models are estimated in each study:
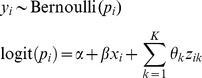
(Model♮_2_)which yields an intercept 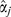, a risk factor-outcome association 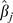, confounder-outcome associations 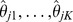 and a within-study covariance matrix 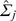 for each study. A summary estimate for the regression coefficients and model intercept can be obtained by extending the bivariate random-effects model from Model 1 into a multivariate generalization [43], [52], [57], [58].



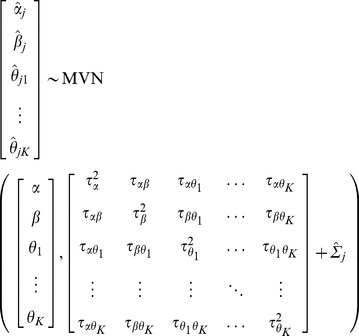
(ModelA)Usually researchers assume zero within-study and between-study correlation, and so perform a separate univariate meta-analysis to each factor-outcome and confounder-outcome association separately; that is Model 2 is fitted for each of the log odds ratio terms separately (Model B).

#### Extended one-stage models

The fully random-effects one-stage model with multiple risk factors is specified as follows:
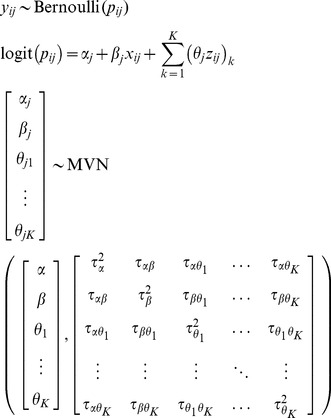
(ModelC)


Alternatively, a *reduced* one-stage model can be estimated by assuming independent random effects for 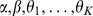, i.e. the off-diagonal terms in Model∼C are set to 0 (Model D).

Finally, it is possible to reduce the number of random effects by stratifying the intercepts and/or predictors for which a summary estimate is not of interest. For example, one-stage *stratified* model that estimates a separate intercept for each study can be achieved as follows:
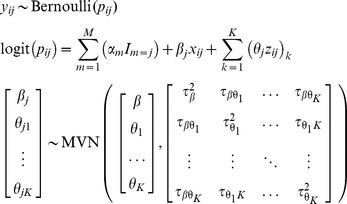
(ModelE)


Stratification on all confounders may, however, not always be feasible due to sample size constraints. For this reason, we generally recommend to model separate intercept terms and to assume random effects for all predictor effects (and hence reduce model complexity by introducing additional assumptions). The underlying rationale is that accurate estimates for confounding parameters are usually not required. Although this simplification may introduce bias in all parameter estimates, baseline risks are likely most affected because they capture all unexplained variation. A non-parametric modeling approach for the intercept terms may thus better accommodate model misspecification.

### Estimation Procedures and Approximations

In the two-stage methods, the first stage model (logistic regression in each study) is estimated using maximum likelihood (ML). In the second stage, the AD meta-analysis models are estimated using, for example, methods of moment (MOM) or restricted maximum likelihood (REML) [21], [52], [54], [59], [60]. This can be implemented in numerous software, with packages such as *lme4* and *mvmeta* in R, *Proc Mixed* in SAS and *mvmeta* in STATA. However, difficulties may arise in the first or second stage estimation. For risk factors that are binary, if zero cell counts occur in some of the included studies (e.g. when all patients with the risk factor presence also have the outcome), the likelihood function may not converge or converges in an unstable factor-outcome association. This problem is also known as (partial) separation [61], [62], and can be overcome by penalization [63]–[67] or adding a continuity correction [68], [69]. A second problem may arise when the number of included studies is small as estimation of between-study covariance may become problematic [43], [52], [70].

One-stage methods involve the estimation of a mixed effects (multilevel) model which is often high dimensional [67]. For this reason, numerical integration is often achieved through approximate methods such as adaptive Gauss-Hermite Quadrature [29], [71]–[73]. Although estimation becomes more precise as the number of quadrature points increases, it often gives rise to computational difficulties and convergence problems [74]. Furthermore, it has been demonstrated that the one-stage method may yield (downwardly) biased variance parameters when studies are small or limited in number [29], [75]–[77]. The one-stage method may also produce downwardly biased coefficient estimates when an incorrect model is specified, for instance when random effects are wrongly assumed [78]. This may increase type-II errors. Although these issues could be reduced by penalization, there is a lack of REML procedures due to the computational difficulty of the second-order Laplace approximation [75].

## Case Studies

In this section, we illustrate the benefits, limitations and differences of one-stage and two-stage methods in the DVT data. For all case studies, in the two-stage models we used MLE in the first stage and MLE, REML or MOM in the second stage. For the one-stage models we used adaptive Gauss-Hermite Quadrature with 1 (Laplacian approximation) and 5 quadrature points.

In the first case study, we performed meta-analyses to estimate the *unadjusted* factor-outcome association for 16 risk factors using each of the models described above, and we examined the obtained log odds ratio (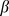), standard error (S.E.), between-study variability (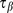) and between-study correlation (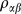). The models considered are: full bivariate two-stage meta-analysis (Model
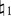
+Model 1), traditional univariate two-stage meta-analysis (Model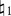 +Model 2), fully random-effects one-stage meta-analysis (Model 3), reduced random-effects one-stage meta-analysis (Model 4) and stratified one-stage meta-analysis (Model 5). For two-stage methods, we penalized the likelihood using Jeffreys invariant prior in datasets with (partial) separation in order to stabilize study-specific estimates [63], [64].

In the second case study, we performed meta-analyses to investigate the risk factor *ddimdich*, adjusted for 3 covariates (*malign*, *surg* and *calfdif3*). Hereto, we estimated the following models: extended full two-stage model (Model
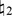
+Model A), extended reduced two-stage model (Model
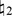
+Model B), extended full one-stage model (Model C), extended reduced one-stage model (Model D) and extended stratified one-stage model (Model E).

For all models, we calculated *p*-values (with 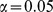) and corresponding 95% confidence intervals for the estimated odds ratios, according to:
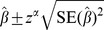
where 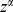 is the 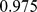 percentile of the standardized normal distribution. Finally, we calculated 95% prediction intervals to indicate a range for the predicted odds ratio in a new study [32], [34]. Assuming the random effects are normally distributed with between-study standard deviation, then an approximate 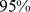 prediction interval for the factor-outcome association in an unspecified study can be obtained as:



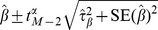
where 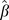 is the estimate of the average factor-outcome association across studies, and 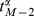 is the 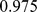 percentile of the Student's 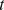 distribution with 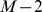 degrees of freedom, where 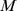 is the number of studies in the meta-analysis.

All models were implemented in R 2.15.1 using Linux Mint 14 Nadia (MATE 64-bit) and incorporated the packages *lme4* (v0.999999-0), *mvmeta* (v0.3.4), *logistf* (v1.10) and *metamisc* (v0.0.4). Additional source code is available in Supporting Information S1.

## Results

### One-stage Versus Two-stage Methods

Results in Table 2 and Table S3 indicate that one- and two-stage methods often yield similar estimates for pooled factor-outcome associations, but importantly not always. For example, for the factor *par* we found an odds ratio of 1.45 (Model∼1 using MLE) versus 1.32 (Model∼5 using MLE). Occasionally, differences led to the one-stage and two-stage models disagreeing upon statistical significance (e.g. *eryt*). These differences mainly occurred when relatively few data were available per study (*coag* and *par*), or relatively few studies were at hand (*eryt* and *ddim*). For instance, the OR of *eryt* was 1.52 (95% CI: 0.93 to 2.47) for the univariate two-stage approach (using DerSimonian and Laird's MOM estimator), versus 1.35 (95% CI 1.03 to 1.77) for the stratified one-stage approach. Furthermore, one-stage and two-stage methods tend to provide different estimates for standard errors and between-study heterogeneity parameters, leading to different prediction intervals. For instance, the prediction interval for the odds ratio of *ddimdich* ranged from 8.65 to 36.20 (Model 2 using MLE), versus 14.24 to 24.17 (Model 5 using MLE). Although usually they give similar results, the univariate two-stage method (Model 2) sometimes obtains different conclusions to the bivariate two-stage method (Model 1). For instance, for *eryt* we respectively found an odds ratio of 1.55 (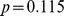) versus 1.38 (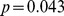) when REML was used as estimation procedure. Finally, the bivariate two-stage method (Model 1) often gives more similar results to the one-stage method. For the factor *eryt*, we found 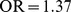 with 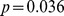 using Model 1 (bivariate two-stage model), versus 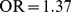 with 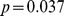 for Model 3 (bivariate one-stage model), 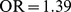 with 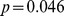 for Model 4 (reduced one-stage model) and 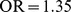 with 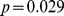 for Model 5 (stratified one-stage model). These estimates were all somewhat different to the results for Model 2 (univariate two-stage MoM) where 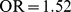 with 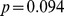.

**Table 2:** Estimated unadjusted factor-outcome associations for the DVT case study.

Risk Factor	Model	Estimation	β	S.E.(β)	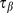	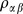	OR	95% CI	95% PI	*p*-value
	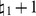	MLE	2.76	0.15	0.30	0.52	15.86	11.73 to 21.45	6.98 to 36.06	<0.001
	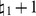	REML	2.78	0.17	0.33	0.28	16.10	11.64 to 22.27	6.48 to 40.00	<0.001
	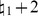	MLE	2.87	0.15	0.25		17.69	13.15 to 23.80	8.65 to 36.20	<0.001
	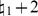	REML	2.89	0.17	0.31		17.97	12.88 to 25.06	7.49 to 47.04	<0.001
ddimdich (8 )	3	MOM	2.89	0.17	0.32		17.98	12.87 to 25.13	7.43 to 43.54	<0.001
	3	MLE 1QP	2.87	0.15	0.28	0.07	17.70	13.14 to 23.86	8.08 to 38.78	<0.001
	3	MLE 5QP	2.85	0.14	0.25	0.58	17.35	13.15 to 22.89	8.62 to 34.91	<0.001
	4	MLE 1QP	2.88	0.15	0.29		17.79	13.15 to 24.07	8.00 to 39.56	<0.001
	4	MLE 5QP	2.85	0.19	0.41		17.37	12.06 to 25.01	5.74 to 52.55	<0.001
	5	MLE 1QP	2.92	0.11	0.00		18.55	15.01 to 22.93	14.24 to 24.17	<0.001
	5	MLE 5QP	2.92	0.11	0.00		18.46	14.94 to 22.81	14.17 to 24.04	<0.001
	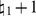	MLE	0.37	0.14	0.26	−0.47	1.45	1.11 to 1.90	0.76 to 2.75	0.007
	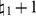	REML	0.38	0.14	10.29	−0.45	1.46	1.10 to 1.93	0.71 to 2.98	0.009
	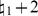	MLE	0.33	0.13	0.23		1.38	1.06 to 1.80	0.76 to 2.51	0.016
	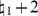	REML	0.33	0.14	0.27		1.39	1.05 to 1.84	0.71 to 2.73	0.020
	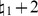	MOM	0.33	0.13	0.24		1.38	1.06 to 1.80	0.76 to 2.52	0.016
par (13 )	3	MLE 1QP	0.32	0.13	0.23	−0.37	1.38	1.07 to 1.79	0.77 to 2.48	0.013
	3	MLE 5QP								
	4	MLE 1QP	0.29	0.13	0.21		1.33	1.03 to 1.71	0.78 to 2.27	0.026
	4	MLE 5QP								
	5	MLE 1QP	0.28	0.13	0.19		1.32	1.03 to 1.70	0.79 to 2.21	0.026
	5	MLE 5QP								
	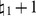	MLE	0.32	0.15	0.10	1.00	1.37	1.02 to 1.84	0.13 to 13.97	0.036
	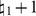	REML	0.32	0.16	0.13	1.00	1.38	1.01 to 1.87	0.10 to 18.23	0.043
	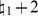	MLE	0.30	0.14	0.00		1.35	1.03 to 1.77	0.23 to 7.87	0.030
	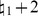	REML	0.44	0.28	0.39		1.55	0.90 to 2.66	0.00 to 664.30	0.115
	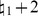	MOM	0.42	0.25	0.33		1.52	0.93 to 2.47	0.01 to 303.63	0.094
eryt (3 )	3	MLE 1QP	0.31	0.15	0.10	1.00	1.37	1.02 to 1.83	0.14 to 13.02	0.037
	3	MLE 5QP	0.31	0.15	0.10	1.00	1.37	1.02 to 1.83	0.14 to 13.04	0.037
	4	MLE 1QP	0.33	0.17	0.14		1.39	1.01 to 1.92	0.09 to 22.31	0.046
	4	MLE 5QP	0.33	0.17	0.14		1.39	1.01 to 1.93	0.09 to 22.62	0.046
	5	MLE 1QP	0.30	0.14	0.00		1.35	1.03 to 1.77	0.23 to 7.80	0.029
	5	MLE 5QP	0.30	0.14	0.00		1.35	1.03 to 1.77	0.23 to 7.80	0.029
	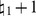	MLE	0.10	0.18	0.20	−1.00	1.11	0.78 to 1.57	0.35 to 3.52	0.574
	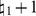	REML	0.10	0.19	0.23	−1.00	1.10	0.76 to 1.60	0.31 to 3.97	0.595
	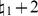	MLE	−0.02	0.15	0.00		0.98	0.73 to 1.31	0.52 to 1.86	0.898
	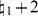	REML	0.02	−0.15	0.00		0.98	0.73 to 1.31	0.52 to 1.86	0.898
	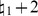	MOM	−0.02	0.15	0.00		0.98	0.73 to 1.31	0.52 to 1.86	0.898
oachst (4† )	3	MLE 1QP	0.08	0.17	0.18	−1.00	1.09	0.77 to 1.53	0.37 to 3.22	0.629
	3	MLE 5QP								
	4	MLE 1QP	−0.03	0.15	0.00		0.98	0.73 to 1.31	0.51 to 1.85	0.866
	4	MLE 5QP								
	5	MLE 1QP	−0.03	0.15	0.00		0.97	0.72 to 1.30	0.51 to 1.84	0.830
	5	MLE 5QP								
	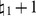	MLE	0.21	0.16	0.22	0.98	1.24	0.91 to 1.68	0.62 to 2.47	0.172
	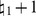	REML	0.22	0.17	0.29	0.82	1.24	0.88 to 1.75	0.52 to 2.95	0.218
	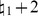	MLE	0.26	0.15	0.15		1.29	0.97 to 1.72	0.75 to 2.23	0.078
	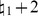	REML	0.26	0.16	0.23		1.29	0.94 to 1.78	0.63 to 2.65	0.116
	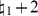	MOM	0.26	0.16	0.21		1.29	0.95 to 1.76	0.66 to 2.51	0.103
coag (7 )	3	MLE 1QP	0.19	0.16	0.23	1.00	1.20	0.88 to 1.64	0.59 to 2.46	0.241
	3	MLE 5QP								
	4	MLE 1QP	0.21	0.16	0.22		1.24	0.90 to 1.69	0.61 to 2.51	0.186
	4	MLE 5QP								
	5	MLE 1QP	0.22	0.13	0.01		1.25	0.97 to 1.61	0.90 to 1.74	0.083
	5	MLE 5QP								

The number between brackets indicates the amount of available studies. Statistical significance (*p*-value), 95% confidence intervals (95% CI) and 95% prediction intervals (95% PI) are given for the odds ratio (OR). For some one-stage models, estimates could not be obtained because the adaptive Gauss-Hermite approximation did not converge.

† Zero-cells occurred in two studies for factor *oachst*.

### Estimation of Correlation between Random Effects

As previously described, only the full one- and two-stage models (Model 1 & Model 3) estimate a parameter for the correlation between random effects. Results in Table 2 demonstrate that these models often yield correlation estimates that are close to +1 or −1, particularly when insufficient data are available and MLE is used. If correlations between random effects are assumed zero (Model 2 & Model 4), we noticed that parameter estimates may considerably change and thereby affect the calculation of *p*-values and prediction intervals. A good example is the unadjusted factor *coag*, where the prediction interval for the OR ranged from 0.62 to 2.47 (Model 1 with MLE) versus 0.75 to 2.23 (Model 2 with MLE), and the corresponding *p*-value decreased from 0.172 to 0.078. Similar findings were obtained for the adjusted analyses (Table 2). Finally, results indicate that the estimated correlation between random effects tends to be less extreme when REML is used (Table 3). The factor *surg* is a good example, as 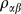 decreased from −0.90 (MLE) to −0.65 (REML).

**Table 3:** Estimated factor-outcome associations in the DVT case study for *ddimdich*, adjusted for *malign*, *surg* and *calfdif3*.

Risk factor	Model	Estimation	β	S.E.(β)	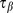	OR	*p*-value
	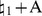	MLE	2.62	0.18	0.40	13.67	<0.001
	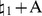	REML	2.64	0.20	0.44	13.80	<0.001
	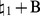	MLE	2.67	0.15	0.25	14.48	<0.001
	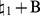	REML	2.69	0.17	0.33	14.75	<0.001
	C	MLE 1QP	2.70	0.18	0.39	14.81	<0.001
ddimdich (10 )	C	MLE 5QP	2.70	0.18	0.40	14.83	<0.001
	D	MLE 1QP	2.67	0.16	0.33	14.42	<0.001
	D	MLE 5QP	2.69	0.14	0.22	14.74	<0.001
	E	MLE 1QP	2.72	0.11	0.00	15.25	<0.001
	E	MLE 5QP	2.72	0.11	0.00	15.25	<0.001

### Estimation of Stratified Models

It is possible to avoid estimating correlation between random effects without assuming independence by using a stratified one-stage model, for example where a separate intercept is estimated for each study (Model 5) and, in the adjusted analyses, where predictors not of key interest are also stratified. Results indicate that the estimation of a separate intercept for each study (Model 5) tends to decrease the standard errors and between-study heterogeneity of factor-outcome associations (unless between-study correlations are +1 or −1). This, in turn, resulted in smaller prediction intervals for estimated odds ratios. For instance, the prediction interval for the unadjusted OR of *ddimdich* ranged from 8.08 to 38.78 (Model 3), versus 14.24 to 24.17 (Model 5).

### Estimation of One-stage Models

One-stage models were estimated with 1 and 5 quadrature points, and sometimes suffered from convergence problems (e.g. *par* and *coag* in Table 2 where positive indefiniteness occurred when 5 quadrature points were used). Possibly, these problems are related to poor model specification. Parameter estimates were similar for 1 and 5 quadrature points in the unadjusted analyses, however, some small differences occurred in the adjusted analyses (e.g. *ddimdich* in Table 3).

## Discussion

We have described several random-effects IPD-MA models that implement a one-stage or two-stage method, where one desires to evaluate a potential causal (risk) factor or predictor of outcome. We detailed how they can be estimated and also extended to adjust for other factors. Despite the conventional belief that one-stage and two-stage methods yield similar conclusions [35]–[37], our empirical investigation shows that this is not always the case. Specifically, we found that different estimates for pooled effects, standard errors, between-study heterogeneity and correlation between random effects can result from choosing a different method (one-stage or two-stage), choosing a different estimation procedure (MLE, REML, MOM, number of quadrature points) and choosing a different model specification (independent random effects, joint random effects, stratified estimation). Although these differences were usually not substantial, in the DVT example they lead to discrepancies concerning the statistical significance of age, duration of symptoms, family history of thrombofilia, presence of erythema, presence of paresis and (dichotomized) D-dimer value.

Thus, importantly the choice of IPD-MA method may actually influence the conclusions about which factors are thought to be risk factors. This makes it desirable to pre-specify in a study protocol what meta-analysis method will be used, to avoid unjustified post-hoc analyses being performed to achieve statistical significance. We generally recommend that the one-stage method should be used. This method models the exact binomial distribution of the data in each study, and does not require a continuity correction when (partial) separation occurs [61]–[64], [67]. The one-stage method may therefore produce more reliable results than the two-stage method when few studies or few subjects per study are available, as the two-stage method incorrectly assumes asymptotic normality (for the log odds ratio estimates from each study) in such scenarios [67]. The one-stage method further facilitates the adjustment for other factors, which is particularly important in non-randomised settings. In addition, one-stage models are more flexible, for example making the implementation of non-linear associations and interactions straightforward [24], [37], [79]–[82]. Finally, stratification in one-stage models avoids the need for estimating correlations between random effects. One can simply estimate study-specific intercepts and slopes and place the random effect only on the factor of interest.

Although we focused on IPD-MA of prognostic factors in this article, the two-stage methods can also be applied when only AD data is available for the included studies. These methods are usually preferred because sharing of IPD is often unfeasible due to, for instance, confidentiality agreements. Results from our empirical example demonstrate that the full two-stage model, which when pooling the AD accounts for heterogeneity of baseline risk and risk factors, and their within-study and between-study correlation, tends to yield most consistent results with the one-stage models. The full two-stage method is a bivariate meta-analysis, which by additionally using the correlation between parameter estimates, is known to have benefits over a univariate me-analysis [43]. The methods presented here could further be extended using methods allowing for the combination of IPD with AD [49], [83], [84]. Potential limitations such as missing data in a subset of studies could be overcome using imputation methods that account for clustering. A Bayesian approach would be the most promising, as it would permit specification of the imputation model alongside the one-stage model, resolving several estimation limitations of the current approaches [32], [85], [86]. Furthermore, Bayesian approaches facilitate sensitivity analyses through adjusting prior specification, and permit the the robustness of fitted models to be evaluated. This is particularly useful when few studies are available and estimated parameters of one- and two-stage models may be severely biased due to estimation difficulties. Future research is needed to evaluate the performance of the described methods, and to compare their accuracy and coverage with Bayesian alternatives.

In summary, the choice of one-stage or two-stage method for performing a random-effects IPD-MA may influence the statistical identification of risk factors (predictors) for a binary outcome. When the number of studies in the meta-analysis are large and the number of events in each study are not few, we agree with Stewart *et al* [37] that a two-stage method will usually suffice. However, we generally recommend that a one-stage IPD-MA method is used as this models the exact binomial distribution, accounts for within-study parameter correlation, offers more flexibility in the model specification and avoids continuity corrections. It is therefore particularly preferable when few studies or few events in some studies are available.

## Supporting Information

Supporting Information S1 **Full model specifications and R code for implementation.**
(GZ) Click here for additional data file.

Table S1 **Overview of the DVT datasets.**
(PDF)

Table S2 **Overview of the variables in the DVT datasets.**
(PDF)

Table S3 **Additional results Case Study.**
(PDF)
