# Supplementary material for: Individual Participant Data Meta-Analysis for a Binary Outcome: One-Stage or Two-Stage?
Source: PLoS One. 2013 Apr 9;8(4):e60650. doi: 10.1371/journal.pone.0060650 (PMC3621872; doi:10.1371/journal.pone.0060650)
Supplement: Table S1 — Overview of the DVT datasets. (PDF) [file pone.0060650.s002.pdf]

Table S1 – Overview of the DVT datasets

All studies were obtained from dr. Geert-Jan Geersing [1, 2].

Study	First author	Year	Country	N	Reference used
1	AMUSE study†	2009	The Netherlands	1 028	CUS
2	R.E.G. Schutgens (AIDA study)	2003	The Netherlands	814	CUS
3	D.R. Anderson (EDIT study)	2000	Canada	153	CUS or venography
4	R.A. Kraaijenhagen	2002	The Netherlands	1 756	CUS
5	D.B. Toll†	2006	The Netherlands	791	CUS
6	D.R. Anderson (EDITED study)	2002	Canada	1 075	CUS or venography
7	C. Kearon	2001	Canada	429	CUS or venography
8	J.L. Elf	2009	Sweden	325	CUS or venography
9	R. Oudega†	2005	The Netherlands	1 295	CUS
10	S.M. Stevens	2004	United States of America	436	CUS
11	P.S. Wells (DIEM study)	2003	Canada	541	CUS
12	S.M. Bates	2003	Canada	550	CUS
13	C. Kearon	2005	Canada	809	CUS or venography

N = number of patients; CUS = compression ultrasonography
† These studies included only primary care patients, other studies included secondary care patients.

References

[1] Geersing GJ. Strategies in suspected venous thrombo-embolism in primary care. Ph.D Thesis. Universiteit Utrecht. Utrecht. The Netherlands. (ISBN 978-94-6108-210-7)

[2] Geersing GJ, Zuithoff NPA, Kearon C, Anderson DR, Elf JL, Bates SM, Hoes AW, Janssen KJM, Kraaijenhagen RA, Oudega R, Schutgens REG, Stevens SM, Wells PS, Moons KGM. Exclusion of Deep Vein Thrombosis using the Wells rule in Primary and Secondary Care; An Individual Patient Data meta-analysis. *Submitted* 2012.
