# Supplementary material for: Individual Participant Data Meta-Analysis for a Binary Outcome: One-Stage or Two-Stage?
Source: PLoS One. 2013 Apr 9;8(4):e60650. doi: 10.1371/journal.pone.0060650 (PMC3621872; doi:10.1371/journal.pone.0060650)
Supplement: Table S2 — Overview of the variables in the DVT datasets. (PDF) [file pone.0060650.s003.pdf]

Table S2 – Overview of the variables in the DVT datasets

Variable	Value	Description
studyid	1–13	the study identification
sex	0	female
	1	male
side	0	side of legpain: left side
	1	side of legpain: right side
	2	side of legpain: both sides
malign	0	no active malignancy
	1	active malignancy
par	0	no paresis
	1	paresis
surg	0	no recent surgery (or bedridden)
	1	recent surgery (or bedridden)
tend	0	no localised tenderness deep venous system
	1	localised tenderness deep venous system
leg	0	entire leg not swollen
	1	entire leg swollen
calfdif3	0	calf difference < 3 cm
	1	calf difference ≥ 3 cm
pit	0	no pitting edema
	1	pitting edema
vein	0	no vein distension
	1	vein distension
altdiagn	0	no alternative diagnosis present
	1	alternative diagnosis present
oachst	0	no oral contraceptives or hst
	1	oral contraceptives or hst used
notraum	0	leg trauma present
	1	no leg trauma present
eryt	0	no erythema
	1	erythema
histdvt	0	no history of previous DVT
	1	history of previous DVT
histpe	0	no history of previous PE
	1	history of previous PE
coag	0	no family history of thrombofilia
	1	family history of thrombofilia
trav	0	no prolonged traveling
	1	prolonged traveling
pregn	0	not pregnant
	1	pregnant
ddimdich	0	D-dimer negative
	1	D-dimer positive
dvt	0	final diagnosis of DVT: no DVT
	1	final diagnosis of DVT: DVT
