# Supplementary material for: Individual Participant Data Meta-Analysis for a Binary Outcome: One-Stage or Two-Stage?
Source: PLoS One. 2013 Apr 9;8(4):e60650. doi: 10.1371/journal.pone.0060650 (PMC3621872; doi:10.1371/journal.pone.0060650)
Supplement: Table S3 — Additional results Case Study. (PDF) [file pone.0060650.s004.pdf]

Table S3 – Additional results Case Study

Estimated unadjusted factor-outcome associations for the DVT case study.

Risk Factor	Model	Estimation	β	S.E.(β)	τ_β	$\rho_{\alpha\beta}$	OR	95% CI	95% PI	p-value
	$\natural_1$+1	MLE	0.88	0.11	0.29	-0.55	2.41	1.96 to 2.97	1.21 to 4.80	< 0.001
	$\natural_1$+1	REML	0.89	0.11	0.32	-0.55	2.42	1.95 to 3.01	1.16 to 5.07	< 0.001
	$\natural_1$+2	MLE	0.87	0.11	0.30		2.38	1.93 to 2.93	1.19 to 4.75	< 0.001
	$\natural_1$+2	REML	0.87	0.11	0.32		2.40	1.92 to 2.98	1.14 to 5.03	< 0.001
	$\natural_1$+2	MOM	0.87	0.11	0.31		2.39	1.93 to 2.96	1.17 to 4.88	< 0.001
pit (13)	3	MLE 1QP	0.87	0.11	0.30	-0.56	2.44	1.98 to 3.01	1.20 to 4.96	< 0.001
	3	MLE 5QP								
	4	MLE 1QP	0.86	0.10	0.30		2.36	1.93 to 2.87	1.24 to 4.48	< 0.001
	4	MLE 5QP								
	5	MLE 1QP	0.85	0.09	0.23		2.33	1.95 to 2.78	1.36 to 4.00	< 0.001
	5	MLE 5QP								
	$\natural_1$+1	MLE	0.36	0.09	0.02	-1.00	1.44	1.22 to 1.70	1.15 to 1.81	< 0.001
	$\natural_1$+1	REML	0.36	0.09	0.02	-1.00	1.44	1.21 to 1.70	1.14 to 1.81	< 0.001
	$\natural_1$+2	MLE	0.36	0.09	0.00		1.43	1.21 to 1.69	1.15 to 1.78	< 0.001
	$\natural_1$+2	REML	0.36	0.09	0.00		1.43	1.21 to 1.69	1.15 to 1.78	< 0.001
	$\natural_1$+2	MOM	0.36	0.09	0.00		1.43	1.21 to 1.69	1.15 to 1.78	< 0.001
notraum (7)	3	MLE 1QP	0.37	0.09	0.02	-1.00	1.45	1.22 to 1.71	1.15 to 1.82	< 0.001
	3	MLE 5QP								
	4	MLE 1QP	0.36	0.09	0.00		1.44	1.22 to 1.70	1.15 to 1.79	< 0.001
	4	MLE 5QP								
	5	MLE 1QP	0.36	0.09	0.00		1.44	1.21 to 1.70	1.15 to 1.79	< 0.001
	5	MLE 5QP								
	$\natural_1$+1	MLE	0.65	0.08	0.11	-1.00	1.91	1.64 to 2.23	1.41 to 2.59	< 0.001
	$\natural_1$+1	REML	0.65	0.08	0.12	-1.00	1.91	1.64 to 2.23	1.40 to 2.61	< 0.001
	$\natural_1$+2	MLE	0.58	0.07	0.00		1.79	1.56 to 2.06	1.53 to 2.10	< 0.001
	$\natural_1$+2	REML	0.58	0.07	0.00		1.79	1.56 to 2.06	1.53 to 2.10	< 0.001
	$\natural_1$+2	MOM	0.59	0.07	0.04		1.80	1.55 to 2.08	1.49 to 2.17	< 0.001
vein (13 †)	3	MLE 1QP	0.64	0.08	0.10	-1.00	1.89	1.62 to 2.19	1.42 to 2.51	< 0.001
	3	MLE 5QP								
	4	MLE 1QP	0.58	0.07	0.00		1.78	1.55 to 2.05	1.52 to 2.09	< 0.001
	4	MLE 5QP								
	5	MLE 1QP	0.57	0.07	0.00		1.78	1.54 to 2.04	1.51 to 2.08	< 0.001
	5	MLE 5QP								
	$\natural_1$+1	MLE	1.22	0.12	0.33	-0.96	3.40	2.67 to 4.33	1.57 to 7.38	< 0.001
	$\natural_1$+1	REML	1.23	0.13	0.35	-0.94	3.41	2.65 to 4.39	1.50 to 7.74	< 0.001
	$\natural_1$+2	MLE	1.18	0.13	0.33		3.27	2.53 to 4.22	1.50 to 7.15	< 0.001
	$\natural_1$+2	REML	1.19	0.14	0.36		3.29	2.52 to 4.28	1.42 to 7.61	< 0.001
	$\natural_1$+2	MOM	1.19	0.14	0.35		3.29	2.52 to 4.28	1.43 to 7.54	< 0.001
malign (13)	3	MLE 1QP	1.22	0.12	0.33	-0.95	3.38	2.66 to 4.30	1.56 to 7.32	< 0.001
	3	MLE 5QP								
	4	MLE 1QP	1.16	0.13	0.31		3.20	2.51 to 4.10	1.53 to 6.71	< 0.001
	4	MLE 5QP								
	5	MLE 1QP	1.17	0.13	0.31		3.22	2.51 to 4.12	1.54 to 6.73	< 0.001
	5	MLE 5QP								
	$\natural_1$+1	MLE	0.40	0.15	0.29	-0.47	1.50	1.11 to 2.02	0.60 to 3.71	0.009
	$\natural_1$+1	REML	0.41	0.17	0.33	-0.45	1.51	1.08 to 2.11	0.53 to 4.28	0.016
	$\natural_1$+2	MLE	0.40	0.16	0.30		1.50	1.10 to 2.04	0.59 to 3.80	0.011
	$\natural_1$+2	REML	0.41	0.18	0.34		1.51	1.07 to 2.14	0.52 to 4.43	0.018
	$\natural_1$+2	MOM	0.42	0.19	0.38		1.53	1.05 to 2.22	0.47 to 5.00	0.026
histdvt (6 ‡)	3	MLE 1QP	0.40	0.15	0.29	-0.47	1.49	1.10 to 2.01	0.60 to 3.69	0.012
	3	MLE 5QP								
	4	MLE 1QP	0.39	0.15	0.28		1.47	1.09 to 1.99	0.60 to 3.59	0.012
	4	MLE 5QP								
	5	MLE 1QP	0.39	0.15	0.28		1.48	1.09 to 2.00	0.60 to 3.62	0.011
	5	MLE 5QP								

The number between brackets indicates the amount of available studies. Statistical significance (p-value), 95% confidence intervals (95% CI) and 95% prediction intervals (95% PI) are given for the odds ratio (OR). For some one-stage models, estimates could not be obtained because the adaptive Gauss-Hermite approximation did not converge.
† Zero-cells occurred in one study for factor *vein*.
‡ One study was excluded because *histdvt*=1 for all subjects.

Risk Factor	Model	Estimation	β	S.E.(β)	τ_β	$\rho_{\alpha\beta}$	OR	95% CI	95% PI	p-value
	$\natural_1$+1	MLE	0.68	0.08	0.07	-0.90	1.97	1.69 to 2.29	1.56 to 2.48	< 0.001
	$\natural_1$+1	REML	0.67	0.08	0.04	-0.65	1.96	1.67 to 2.30	1.47 to 2.63	< 0.001
	$\natural_1$+2	MLE	0.65	0.08	0.04		1.91	1.65 to 2.21	1.59 to 2.29	< 0.001
	$\natural_1$+2	REML	0.65	0.08	0.09		1.91	1.63 to 2.23	1.47 to 2.47	< 0.001
	$\natural_1$+2	MOM	0.65	0.08	0.09		1.91	1.63 to 2.23	1.46 to 2.49	< 0.001
surg (13)	3	MLE 1QP	0.66	0.08	0.07	-0.77	1.94	1.67 to 2.26	1.53 to 2.46	< 0.001
	3	MLE 5QP								
	4	MLE 1QP	0.64	0.08	0.05		1.89	1.63 to 2.20	1.56 to 2.30	< 0.001
	4	MLE 5QP								
	5	MLE 1QP	0.64	0.07	0.00		1.89	1.64 to 2.19	1.61 to 2.23	< 0.001
	5	MLE 5QP								
	$\natural_1$+1	MLE	0.56	0.12	0.35	-0.23	1.75	1.39 to 2.21	0.77 to 3.99	< 0.001
	$\natural_1$+1	REML	0.56	0.12	0.37	-0.24	1.76	1.38 to 2.24	0.74 to 4.17	< 0.001
	$\natural_1$+2	MLE	0.56	0.12	0.36		1.76	1.39 to 2.23	0.76 to 4.05	< 0.001
	$\natural_1$+2	REML	0.57	0.12	0.38		1.76	1.38 to 2.25	0.73 to 4.22	< 0.001
	$\natural_1$+2	MOM	0.57	0.14	0.42		1.76	1.35 to 2.30	0.67 to 4.66	< 0.001
tend (13)	3	MLE 1QP	0.57	0.12	0.35	-0.23	1.76	1.40 to 2.22	0.78 to 4.01	< 0.001
	3	MLE 5QP								
	4	MLE 1QP	0.55	0.12	0.34		1.74	1.39 to 2.18	0.79 to 3.85	< 0.001
	4	MLE 5QP								
	5	MLE 1QP	0.56	0.11	0.31		1.75	1.41 to 2.16	0.85 to 3.61	< 0.001
	5	MLE 5QP								
	$\natural_1$+1	MLE	1.15	0.12	0.35	-0.31	3.16	2.48 to 4.01	1.38 to 7.19	< 0.001
	$\natural_1$+1	REML	1.15	0.13	0.38	-0.31	3.17	2.47 to 4.08	1.32 to 7.64	< 0.001
	$\natural_1$+2	MLE	1.15	0.12	0.36		3.15	2.47 to 4.01	1.37 to 7.26	< 0.001
	$\natural_1$+2	REML	1.15	0.13	0.38		3.17	2.46 to 4.08	1.30 to 7.72	< 0.001
	$\natural_1$+2	MOM	1.15	0.12	0.39		3.17	2.45 to 4.10	1.29 to 7.83	< 0.001
leg (13)	3	MLE 1QP	1.15	0.12	0.36	-0.32	3.17	2.49 to 4.04	1.37 to 7.33	< 0.001
	3	MLE 5QP								
	4	MLE 1QP	1.14	0.12	0.35		3.11	2.46 to 3.95	1.37 to 7.07	< 0.001
	4	MLE 5QP								
	5	MLE 1QP	1.14	0.11	0.32		3.12	2.49 to 3.91	1.46 to 6.64	< 0.001
	5	MLE 5QP								
	$\natural_1$+1	MLE	1.39	0.08	0.18	-0.35	4.02	3.44 to 4.70	2.60 to 6.20	< 0.001
	$\natural_1$+1	REML	1.39	0.08	0.20	-0.34	4.02	3.42 to 4.73	2.51 to 6.43	< 0.001
	$\natural_1$+2	MLE	1.38	0.08	0.18		3.97	3.39 to 4.65	2.56 to 6.15	< 0.001
	$\natural_1$+2	REML	1.38	0.08	0.20		3.98	3.38 to 4.68	2.48 to 6.38	< 0.001
	$\natural_1$+2	MOM	1.38	0.08	0.20		3.98	3.37 to 4.69	2.46 to 6.44	< 0.001
calfdif3 (13)	3	MLE 1QP	1.39	0.08	0.20	-0.34	4.03	3.45 to 4.71	2.61 to 6.22	< 0.001
	3	MLE 5QP								
	4	MLE 1QP	1.38	0.08	0.17		3.96	3.40 to 4.61	2.62 to 5.98	< 0.001
	4	MLE 5QP								
	5	MLE 1QP	1.38	0.07	0.12		3.96	3.47 to 4.52	2.95 to 5.32	< 0.001
	5	MLE 5QP								
	$\natural_1$+1	MLE	-1.35	0.19	0.63	-0.45	0.26	0.18 to 0.38	0.06 to 1.11	< 0.001
	$\natural_1$+1	REML	-1.35	0.20	0.66	-0.45	0.26	0.17 to 0.38	0.06 to 1.19	< 0.001
	$\natural_1$+2	MLE	-1.37	0.19	0.63		0.25	0.17 to 0.37	0.06 to 1.09	< 0.001
	$\natural_1$+2	REML	-1.37	0.20	0.66		0.25	0.17 to 0.38	0.06 to 1.16	< 0.001
	$\natural_1$+2	MOM	-1.37	0.20	0.64		0.25	0.17 to 0.37	0.06 to 1.10	< 0.001
altdiagn (13)	3	MLE 1QP	-1.37	0.19	0.63	-0.44	0.25	0.17 to 0.37	0.06 to 1.09	< 0.001
	3	MLE 5QP								
	4	MLE 1QP	-1.40	0.19	0.62		0.25	0.17 to 0.36	0.06 to 1.03	< 0.001
	4	MLE 5QP								
	5	MLE 1QP	-1.40	0.19	0.62		0.25	0.17 to 0.36	0.06 to 1.03	< 0.001
	5	MLE 5QP								
	$\natural_1$+1	MLE	0.48	0.05	0.03	1.00	1.62	1.46 to 1.80	1.42 to 1.85	< 0.001
	$\natural_1$+1	REML	0.48	0.05	0.03	1.00	1.62	1.46 to 1.80	1.42 to 1.85	< 0.001
	$\natural_1$+2	MLE	0.50	0.05	0.00		1.64	1.48 to 1.82	1.46 to 1.85	< 0.001
	$\natural_1$+2	REML	0.50	0.05	0.00		1.64	1.48 to 1.82	1.46 to 1.85	< 0.001
	$\natural_1$+2	MOM	0.50	0.05	0.00		1.64	1.48 to 1.82	1.46 to 1.85	< 0.001
sex (13)	3	MLE 1QP	0.48	0.05	0.03		1.62	1.46 to 1.80	1.42 to 1.85	< 0.001
	3	MLE 5QP								
	4	MLE 1QP	0.50	0.05	0.00		1.64	1.48 to 1.82	1.46 to 1.84	< 0.001
	4	MLE 5QP								
	5	MLE 1QP	0.50	0.05	0.00		1.64	1.48 to 1.82	1.46 to 1.85	< 0.001
	5	MLE 5QP								

The number between brackets indicates the amount of available studies. Statistical significance (p-value), 95% confidence intervals (95% CI) and 95% prediction intervals (95% PI) are given for the odds ratio (OR). For some one-stage models, estimates could not be obtained because the adaptive Gauss-Hermite approximation did not converge.
